# Integrative dynamics of cell wall architecture and plant growth under salt stress

**DOI:** 10.3389/fpls.2025.1644412

**Published:** 2025-07-30

**Authors:** Faheem Tariq, Changle Ma, Shuangshuang Zhao

**Affiliations:** ^1^ Shandong Provincial Key Laboratory of Plant Stress, College of Life Sciences, Shandong Normal University, Jinan, China; ^2^ State Key Laboratory of Crop Biology, College of Agronomy, Shandong Agricultural University, Tai’an, Shandong, China

**Keywords:** salt stress, cell wall composition, plant cell anisotropy, cell wall integrity, cell growth

## Abstract

Salt stress is a major challenge to agricultural productivity and can adversely affect plant growth and development. This review examines the interaction between cell wall architecture and plant tolerance to salt stress, focusing on the mechanisms underlying growth, remodeling, and anisotropic morphogenesis. It further elucidates how the cell wall’s composition, structure, and mechanical properties affect osmotic balance, ion transport, and physiological responses to salinity in plants. Key strategies for adaptation to stress, including the synthesis of osmoprotectants and alterations in cell wall polysaccharides, are discussed to understand their role in cell integrity and expansion under salt conditions. In addition, the review emphasizes the dynamic remodeling of the cell wall, which promotes anisotropic growth patterns necessary to maintain plant structure and function under environmental stresses. Based on the current research, this review highlights potential pathways to enhance plant adaptation to salinity through targeted manipulation of cell wall properties, providing insights for future biotechnological applications to improve crop performance in a saline environment.

## 1 Introduction

In natural environments, plants are constantly exposed to various type of environmental stresses, both abiotic and biotic, that can significantly affect plant growth, development, and productivity (Du et al., 2024). Salt is one of the most common and harmful abiotic stress, which leading to decreased crop yield as it inhibits plant growth and limits important cellular processes (Yadav et al., 2020). Salt stress is estimated to affect over 6% of the world’s land area, approximately 800 million hectares (Mustafa et al., 2020). Salinity exhibits a huge impact on agriculture, causing substantial economic losses annually due to reduced crop yields (Chele et al., 2021). Furthermore, the irrigated agricultural land is frequently vulnerable due to salinization, which is predicted to worsen over time and potentially impact as much as 50% of irrigated land by 2050 (Singh, 2022). Salt stress typically begins with the accumulation of sodium ions (Na^+^) in the root zone, which creates osmotic imbalance and disrupts cellular hydration, thereby inhibiting water uptake (Atta et al., 2023; Héreil et al., 2024). The excess Na^+^ enters plant cells primarily through non-selective cation channels and competitive uptake mechanisms, displacing essential K^+^ ions and disrupting enzymatic activity and membrane integrity (Estrada et al., 2023; Zhou et al., 2024). Intracellular Na^+^ accumulation alters the ionic equilibrium and raises osmotic pressure, leading to metabolic hinderance, oxidative stress via reactive oxygen species (ROS), and reduced photosynthesis (Zhao et al., 2020).

Salt stress impacts various cellular structures, including the cell wall, which plays a crucial role in maintaining plant growth and development. Cell wall forms the first layer of the plant cell. The changes caused as a result of salt stress extend to the cell wall, which is a vital component of plant cells, and plays a key role in regulating growth, maintaining cell shape, and responding to environmental stresses (Cosgrove, 2022). The cell wall’s ability to adapt and remodel in response to stress is crucial to plant survival under salinity. Particularly, the accumulation of Na^+^ ions in the apoplast (the space outside the cell membrane) can disrupt cell wall loosening by interacting with negatively charged cell wall polymers, such as pectin and hemicelluloses. This interaction alters the pH and hinders the plant’s ability to extend its cell walls, thereby restricting growth (Dabravolski and Isayenkov, 2023).

Salt stress impacts cell wall structure not only chemically but also mechanically, disrupting anisotropic growth, which normally allows cells to expand more in one direction such as elongating hypocotyls and roots while maintaining directional stability (Colin et al., 2022). This process relies on the orientation of cellulose microfibrils, guided by cortical microtubules, which direct expansion along preferred axes. Under salt stress, there is a transient depolymerization of microtubules and removal of cell‐wall synthesis complexes (CESAs) from the plasma membrane. However, during favorable condition the microtubules and CESAs reassemble, that is facilitated by proteins like CC1/2 and SP2L, to re-establish cellulose orientation for growth recovery (Yu et al., 2022). Additionally, salt exposure often shifts growth patterns from anisotropic to radial expansion, notably in epidermal cells, a morphological change that compromises organ form. The SP2L-mediated reorientation of microtubules has been shown to induce such radial cell expansion in root transition zones, altering directional growth (Van de Peer et al., 2021; Yu et al., 2022).

**Figure 1:**
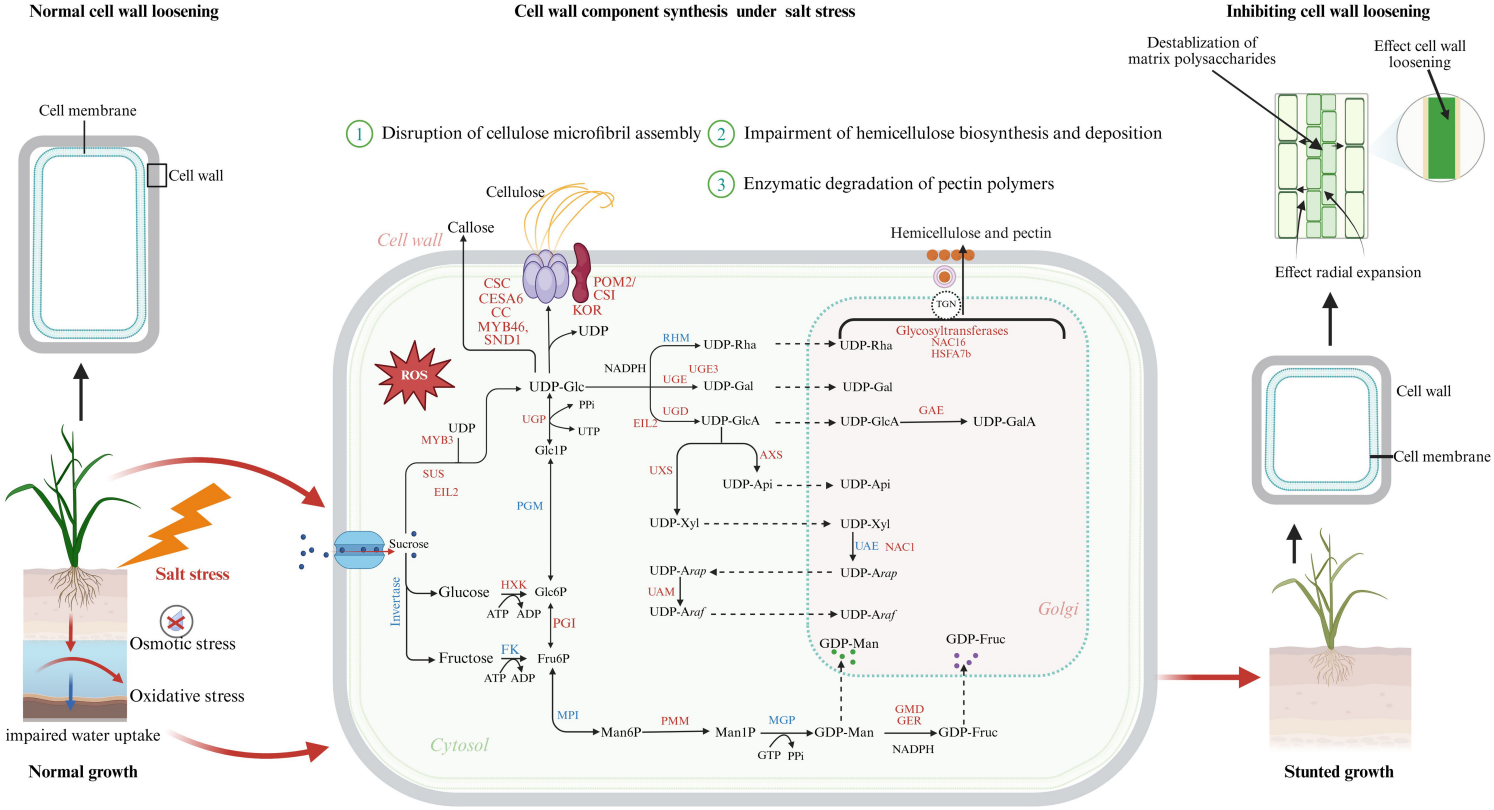
Overview of the effects of salt stress on plant cell wall integrity, loosening, and component biosynthesis. Salt stress in plants triggers both osmotic and oxidative stress, resulting in impaired water uptake and excessive generation of reactive oxygen species (ROS). These ROS contribute to structural damage and destabilization of matrix polysaccharides, ultimately inhibiting cell wall loosening and radial expansion. Consequently, the biosynthesis and stability of major wall components are adversely affected: cellulose becomes weakened, hemicellulose synthesis is disrupted, pectin is degraded, and lignin biosynthesis is suppressed. The intracellular sugar metabolism and biosynthetic pathways contributing to the formation of cellulose, hemicellulose, and pectin. Red-labeled genes and enzymes represent transcriptional or post-transcriptional upregulation under salt stress, including transcription factors (MYB46, SND1, NAC1, NAC16, HSFA7b, EIL2, MYB3) and key biosynthetic enzymes (UGE3, UGD, UGP, UXS, GMD, GER, GAE), which drive stress-induced remodeling of cell wall metabolism. Under normal conditions (left panel), cell wall-loosening enzymes such as XTHs and expansins facilitate expansion and growth, while salt stress (right panel) disrupts these processes, leading to reduced wall plasticity and stunted plant development. The figure integrates metabolic, transcriptional, and biophysical responses, providing a comprehensive view of how salt stress alters plant cell wall dynamics and growth.

The plant cell wall is a dynamic, multifunctional structure composed of polysaccharides (cellulose, hemicellulose, pectin), structural proteins, and lignin, which collectively provide mechanical support while modulating the movement of ions, water, and signaling molecules across the cell boundary (Dokka et al., 2024). The cell wall plays a critical role in determining how plants respond to external stresses, including salinity (Dabravolski and Isayenkov, 2023). Plants have developed several defense mechanisms against the harmful consequences of salt stress, including cell wall remodeling. Through this remodeling, plants preserve cellular integrity, maintain ion homeostasis, and alter developmental patterns to avoid high salinity regions (Oliveira et al., 2020). Moreover, during salt stress, plants activate a series of biochemical pathways that modify cell wall components, such as pectin, hemicelluloses, and cellulose (**Figure 1**). These modifications allow the cell wall to maintain its integrity and continue supporting cell growth, even under adverse conditions (Dabravolski and Isayenkov, 2023).

This review focuses on the complex relationship between salt stress and plant cell wall architecture, with a focus on understanding the physiological, biochemical, and mechanical processes involved in cell wall remodeling. By examining these processes, this review aims to shed light on how plants utilize cell wall modifications to enhance their tolerance to salt stress, thus providing a foundation for developing strategies to improve crop resilience in saline environments.

## 2 Role of cell wall architecture in salt stress

The cell wall serves as a complex extracellular matrix surrounding most of plant cells and exhibits extreme tensile strength and extensibility. Its architecture is crucial, as it supports various aspects of plant growth and development. The cell wall frequently serves as a robust yet pliable structure, which is perpetually remodeled to direct the process of cellular expansion. The architectural composition of the plant cell wall is diverse and species-specific. The structure and composition of the plant cell wall vary between monocots and dicots. While both share key components such as cellulose, hemicelluloses, and pectin, dicot primary walls are typically rich in pectin and xyloglucans, whereas monocot (especially grasses) walls contain more glucuronoarabinoxylan and mixed-linkage glucans with lower pectin content (Cosgrove, 2022). This distinction is critical when considering cell wall remodeling under salt stress, as the biochemical pathways involved differ between plant types. While recognizing these structural distinctions between monocot and dicot cell walls, the present review aims to integrate current knowledge across both plant types to provide a comprehensive understanding of how salt stress modulates cell wall components and architecture (**Table 1**).

**Table 1:** Roles of cell wall different component under salt stress.

Component	Structure and composition	Functional role in normal conditions	Response under salt stress	References
Cellulose	Linear β-1,4-glucan chains forming microfibrils	Provides tensile strength and structural support	upregulated to reinforce wall rigidity	(Cosgrove, 2022)
Hemicellulose	Branched polysaccharides (e.g., xyloglucans in dicots, glucuronoarabinoxylan in monocots)	Cross-links cellulose microfibrils, modulates flexibility	Modified to adjust wall extensibility; monocots favor GAX cross-linking via ferulates	(Scheller and Ulvskov, 2010; Rui and Dinneny, 2020)
Pectin	Complex polysaccharides rich in galacturonic acid; highly methyl-esterified in dicots	Maintains wall porosity and hydration; mediates cell adhesion	De-esterified pectin cross-links with Ca^2+^ to restrict Na^+^ intrusion and strengthen walls	(Geilfus, 2017)
Lignin	Phenolic polymer derived from monolignols; mainly in secondary walls	Adds rigidity and hydrophobicity; restricts pathogen entry	Salt stress induces lignification to prevent cell collapse and ion intrusion	(Xu et al., 2020)
Structural proteins	Hydroxyproline-rich glycoproteins (e.g., extensins, proline-rich proteins)	Strengthens wall structure; anchors pectin and cellulose matrices	Extensin arabinosylation helps stabilize wall and limit cell wall loosening	(Velasquez et al., 2011)
Arabinogalactan proteins	Highly branched glycoproteins with arabinose and galactose side chains	Involved in cell expansion, signaling, and development	AGPs accumulate at the plasma membrane and apoplast to modulate ion buffering and cell signaling	(Zhao et al., 2019)

### 2.1 Cellulose

In terrestrial vascular plants, the cell wall is primarily composed of cellulose, a homopolymer made up of repeating glucose subunits linked by β (1-4) bonds (Ellinger and Voigt, 2014; Schneider et al., 2016). Cellulose plays a crucial role as the main load-bearing component of the cell wall, providing mechanical support. The synthesis of cellulose is an active process, with its synthesis trajectory guided by microtubules within the plant cell. During this process, a heterotrimeric rosette structure known as CesA interacts with Plant Oligosaccharide Binding Protein 2/Cellulose Synthase Interacting Protein 1 (POM2/CSI1) to facilitate cellulose production (Bringmann et al., 2012). The role of CesA in cellulose synthesis has been explored in previous studies (Joshi and Mansfield, 2007; Lampugnani et al., 2018). A mutation in the catalytic domain of CESA6 results in reduced cellulose content in plants (Huang et al., 2023). Although there is still a lack of extensive experimental evidence showing how CesA genes function under salt stress conditions in plants (Heyndrickx and Vandepoele, 2012). General Control Non-repressed Protein 5 (GCN5) plays a key role in regulating cellulose synthesis in salt-sensitive *Arabidopsis thaliana* by modulating the transcription of cellulose biosynthetic genes, thereby contributing to enhanced salt stress tolerance (Zheng et al., 2019). Mutants deficient in cellulose, such as *cesa6*, *pom2/csi1*, and the *Companion of Cellulose Synthases* (*CC*) mutant, display heightened sensitivity to salt stress. These mutants display a sensitive phenotype to salt stress. This increased sensitivity is primarily due to impaired cellulose biosynthesis, which compromises cell wall integrity and reduces the plant’s ability to cope with environmental stressors (Endler et al., 2015; Zhang et al., 2016a).

Salt stress inhibits cellulose synthesis, leading to altered cell wall structure and impaired plant growth (**Table 2**). This inhibition is partly due to the depolymerization of cortical microtubules (MTs), which disrupts the delivery and orientation of the cellulose synthase complex (CSC)—a plasma membrane-localized, multi-subunit protein complex responsible for polymerizing UDP-glucose into β-1,4-glucan chains that form cellulose microfibrils (Wang et al., 2007). The coordination between CSC and MTs is facilitated by the CC proteins, which act as molecular bridges by simultaneously binding to CSC and MTs, thereby aligning cellulose deposition with cytoskeletal dynamics. However, the precise feedback mechanism by which MTs rely on CSC—and ultimately the cell wall—for stress signal perception remains unresolved.

**Table 2:** Genes involved in cellulose biosynthesis, modification, and their roles under salt stress conditions.

Gene family	Gene name	Gene ID	Function	Role in Salt Stress	Reference
Cellulose Synthase (CesA)	CESA1, CESA3, CESA4, CESA6	AT4G32410, AT5G05170, AT5G44030, AT5G64740	Cellulose biosynthesis in the primary cell wall	Provides tolerance against salt stress by maintaining cell wall rigidity and flexibility	(Persson et al., 2007; Li et al., 2017)
	CESA7, CESA8	AT5G17420, AT4G18780	Cellulose biosynthesis in the secondary cell wall	Reinforces the cell wall structure under salt stress, improving mechanical properties	(Taylor-Teeples et al., 2015; Du et al., 2022)
Glucan Synthase-Like (GSL)	GSL1	AT4G04970	Callose biosynthesis at plasmodesmata and during cell division	Plays a role in maintaining cellular integrity under salt stress by regulating callose deposition	(Shikanai et al., 2022)
	GSL5	AT4G03550	Callose deposition in response to pathogen infection and abiotic stress	Enhances callose deposition under salt stress, contributing to cell wall reinforcement	(Saatian et al., 2023)
	GSL12	AT5G13000	Callose deposition during pollen development	Regulates callose deposition during salt stress, aiding in stress response and cellular protection	(Motomura et al., 2021)

Moreover, proper cellulose biosynthesis under salinity is regulated by protein glycosylation. The N-glycosylation pathway in the endoplasmic reticulum (ER) modulates salt tolerance and CSC functionality in a mature N-glycan-dependent manner. In particular, complex N-glycans are essential for the activity of KORRIGAN 1 (KOR1, also known as RADIALLY SWOLLEN2 or RSW2), a membrane-bound endo-1,4-β-glucanase that contributes to cellulose microfibril elongation (Kang et al., 2008). Additionally, transcriptomic analyses in the salt-tolerant *Dendrobium officinale* revealed that *CELLULOSE SYNTHASE-LIKE A* (*CSLA*) genes are upregulated under salt stress and are involved in the biosynthesis of mannan-type hemicelluloses, highlighting the broader impact of salt stress on non-cellulosic polysaccharide production (He et al., 2015).

### 2.2 Hemicellulose

Hemicellulose composition in the cell wall varies between species but is mainly composed of a polysaccharide backbone linked by β (1→4) linkages. Glucans with β (1→3, 1→4) links are grouped with xyloglucans, xylans, glucomannans, mannans, and other similar families (Scheller and Ulvskov, 2010). In dicotyledonous plants, xyloglucans (XyGs) typically constitute 20–25% of the primary cell wall and serve as the major hemicellulose component. In contrast, monocots possess type II primary walls in which glucuronoarabinoxylan dominate, and XyGs account for only 1–5% of total wall polysaccharides (Gigli-Bisceglia et al., 2020; Alvarez et al., 2024).

In dicots, XyGs form tightly bound complexes with cellulose, creating “hotspots” that play a central role in regulating wall mechanics. These hotspots bear localized mechanical stress during cell expansion and wall loosening, while pectins assist by modulating wall porosity and promoting cell elongation and division (Cosgrove and Jarvis, 2012). However, despite the structural significance of XyG-cellulose interactions, the precise biochemical impact of these hotspots on the mechanical properties of the cell wall remains incompletely understood and requires further investigation.

XyGs consists of a β-(1,4)-linked glucose backbone with α-(1,6)-linked xylosyl side chains. In some cases, these side chains are further modified with fucose or galactose, resulting in a complex, branched structure (Schultink et al., 2014; Pauly and Keegstra, 2016). The XyGs are a spacer polymer in the primary cell wall (Thompson, 2005; Anderson et al., 2010). Xylan is widely known as β-(1-4)-linked xylose residue decorated by glucuronic acid to form glucuroxylan. Xylan is mainly accumulated in the secondary cell wall; similarly, it is also present in some algae and monocots. The biosynthesis of XyGs takes place in the Golgi apparatus via glycan synthase and glycosyltransferases. XyGs are then exported to the plasma membrane through packing in vesicles and released into the extracellular matrix, where modification takes place via multiple extracellular enzymes, and eventually become part of the cell wall (Pauly and Keegstra, 2016).

Many studies have been conducted to better understand how hemicellulose, specifically xyloglucan, functions under salt stress (**Table 3**). Under salinity stress, xyloglucan is a metabolic inducer that triggers different physiological responses (Páez-Watson et al., 2020). The xyloglucan endotransglucosylase/hydrolase (XTH) enzyme plays a key role in altering cell wall morphology by cleaving and restructuring XyGs, thereby facilitating the reorganization of the cell wall matrix. The reduced end of XyG is then linked to the non-reducing end of another XyG polymer or oligomer. The gene *CaXTH3* is involved in cell wall remodeling by accumulating starch content, which enables the plant mesophyll cell to retain humidity, and is actively involved in numerous cellular processes and salinity stress tolerance in transgenic *Arabidopsis* seedlings (Cho et al., 2006; González-Pérez et al., 2018). Additionally, the involvement of *XTH19* and *XTH23* in the brassinosteroid (BR) signaling elucidates how the BES1-dependent pathway affects lateral root development under salinity stress (Xu et al., 2020). Furthermore, XTH30 negatively regulates salt tolerance by promoting microtubule depolymerization and reducing crystalline cellulose deposition (Yan et al., 2019). Additionally, under salinity stress, the hemicellulose in the cell wall of *Artemisia annua* shows an increase in xylose content (Correa-Ferreira et al., 2019). The xyloglucan plays a significantly important role in defining the cell shape by regulating the cellulose microfibril’s loosening and tightening events during cell growth and maturation. However, little is known about how salt stress impacts the XyG structure and thus plant growth and development.

**Table 3:** Hemicellulose-related genes involved in cell wall synthesis and their roles under salt stress conditions.

Gene	Gene ID	Hemicellulose type	Function	Role in Salt Stress	Reference
IRX9	AT2G37090	Xylan	The biosynthesis of the xylan backbone	Preserves cell wall strength by maintaining xylan structure under osmotic stress	(Bauer et al., 2006)
IRX14	AT4G36890	Xylan	Facilitates xylan backbone biosynthesis	Contributes to cell wall integrity during salt stress	(Keppler and Showalter, 2010)
IRX10	AT1G27440	Xylan	Critical for elongating the xylan chain	Ensures mechanical properties of cell wall during osmotic stress	(Brown et al., 2009; Wu et al., 2009)
IRX8	AT5G54690	Xylan	Xylan formation and its integration into the cell wall	Modulates cell wall structure and signals stress response pathways	(Persson et al., 2007)
IRX12	AT2G38080	Xylan	Arabinose substitution of xylan	Contributes to hemicellulose complexity, aiding cell wall plasticity under salt stress	(Taylor-Teeples et al., 2015)
CSLA1, CSLA2, CSLA3, CSLA7, CSLA9	AT4G16590, AT5G22740, AT1G23480, AT2G35650, AT5G03760	Glucomannan and xyloglucan	Synthesis of glucomannan and xyloglucan, key polysaccharides in the cell wall	Enhances cell wall stability during salt stress by maintaining proper interaction with cellulose	(Goubet et al., 2009)
CSLB1, CSLB2	AT2G32610, AT2G32620	Hemicellulose (likely related to xyloglucan modification)	Likely involved in modifying hemicellulose structures in the cell wall	Contributes to cell wall resilience under salt stress	(Dekkers et al., 2013)
CSLC4, CSLC5, CSLC6, CSLC12	AT3G28180, AT4G31590, AT3G07330, AT4G07960	Xyloglucan	Biosynthesis of xyloglucan, which cross-links with cellulose for matrix flexibility	Maintains cell wall integrity and flexibility under osmotic stress caused by salt stress	(Kim et al., 2020)
CSLD1, CSLD2, CSLD3, CSLD4	AT2G33100, AT5G16910, AT3G03050, AT4G38190	Xyloglucan, glucomannan (likely related to secondary cell walls)	Likely involved in secondary cell wall biosynthesis, interacting with cellulose	Strengthens secondary cell walls, enhancing flexibility during salt stress	(Bernal et al., 2007, 2008; Zhao et al., 2022)
MUR3	AT2G20370	Xyloglucan	Xyloglucan side-chain modification	Adjusts cell wall flexibility, supporting osmotic adjustment under salinity	(Li et al., 2013)
XLT2	AT5G62220	Xyloglucan	Modifies xyloglucan backbone, aiding in cellulose interaction	Enhances cell wall flexibility to adapt to salt-induced osmotic changes	(De Caroli et al., 2014)
PARVUS	AT1G19300	Glucuronoxylan	Glucuronoxylan biosynthesis in secondary cell walls	Maintains secondary cell wall structure under saline conditions	(Shao et al., 2004)
UXS1	AT3G53520	Glucuronoxylan	Produces UDP-xylose, a precursor for glucuronoxylan	Regulates hemicellulose content and water balance during salt stress	(Kuang et al., 2016)

### 2.3 Pectin

Pectin is a major component of the primary cell wall and the most diverse polysaccharide. Backbone of pectin consists of α-(1,4)-linked galacturonic acid (GalA), that are classified into three types: complex branched rhamnogalacturonan II (RG II), homogalacturonan (unbranched HG), and rhamnose-alternated (RG I), i.e., rhamnogalacturonan I α-(1,4) GalA and α-(1,2) rhamnose (Atmodjo et al., 2013; Amos et al., 2018). The pectin structure is strengthened by Ca^2+^-bridged HG and dimerized RG-II, which is also linked to boron ions (B^3+^). These interactions contribute to the strength of the primary cell wall in terrestrial plants (Liu et al., 2012; Oda and Fukuda, 2012; Kim et al., 2020). Pectin interacts with cellulose at RG-I blocks (arabinan galactan side chains) (Zykwinska et al., 2005). RG-II bonding is reinforced by the Ca^2+^ pectate bond. HG biosynthesis is mediated by galacturonosyl transferases (GAUTs). A detailed review of pectin biosynthesis and structure has been published by Zhang et al. (2021).

Pectin in the cell wall plays a vital role during cell proliferation and plant growth by providing flexibility (Peaucelle et al., 2012). FERONIA (FER) receptor kinases localized at the plasma membrane act as a sensor to sense pectin-associated cell wall damage during salinity stress (Feng et al., 2018; Verger and Hamant, 2018). Pectin methylesterase inhibitor (PMEI) negatively regulates salt resistance and reduces primary root growth (Jithesh et al., 2012). PMEIs maintain a high degree of pectin methyl esterification by inhibiting the activity of pectin methylesterases (PMEs). This suppression of PME activity limits the formation of calcium-mediated cross-links between homogalacturonan chains, thereby preserving cell wall plasticity. Such regulation is vital for maintaining primary root growth under saline conditions, as increased pectin methyl esterification enhances cell wall loosening and facilitates cell elongation and expansion (Peaucelle et al., 2011). The dynamic modulation of pectin structure through the PME–PMEI balance demonstrates a critical mechanism by which plants sustain root architecture and cellular integrity under abiotic stress.

Under salinity stress, the disruption of pectin structure plays a crucial role in root growth regulation (Chen et al., 2018). In developing plant cells, invasive Na^+^ competes with Ca^2+^ for binding sites on galacturonic acid residues in homogalacturonan, destabilizing the Ca^2+^-crosslinked structure. Similarly, the borate-crosslinked structure of GCN5 is also disrupted, weakening the integrity of the cell wall (Munarin et al., 2012). This structural compromise is sensed by FER, a cell wall integrity (CWI) sensor protein, which activates signaling pathways to mitigate the damage and restore growth (Feng et al., 2018). PMEIs, including salt tolerant *Chorispora bungeana* CbPMEI1, regulate PME activity, preventing excessive pectin demethylesterification that could otherwise increase the vulnerability of pectin to degradation under salt stress. Levels of pectin in the cell wall are modified by TRICHOME SPECIFIC DEFECTIVE 2 (TSD2) in rice, when exposed to salinity stress (Fang et al., 2019). Pectin methylesterase 31 (PME31), localized at the plasma membrane, positively modulates salt stress tolerance in Arabidopsis by decreasing RD29A, DREB2A, and RD29B expression, thereby reducing stress-induced ABA signaling and osmotic stress responses, ultimately contributing to cell wall stability and adaptive growth under saline conditions (Yan et al., 2018). Pectin plays a key role in regulating cell growth, and under salt stress, cellulose content increases while pectin levels decrease (**Table 4**). These findings suggest that salt stress enhances the rigidity of the cell wall, which inhibits root growth (An et al., 2014).

**Table 4:** Genes involved in pectin synthesis and their roles in salt stress adaptation.

Gene Family	Gene Name/Distributed Gene Family	Gene ID	Function	Role under Salt Stress	Reference
Galacturonic Acid Synthesis	GAUT1, GAUT7	AT1G18580, AT2G38650	Synthesis of homogalacturonan, maintaining cell wall integrity and structure.	Regulates water retention, helping plants maintain cell shape and turgor pressure under saline conditions.	(Atmodjo et al., 2011; Lund et al., 2020)
Xylogalacturonan Synthesis	XGD1	AT5G33290	Synthesis of xylogalacturonan, a pectin type that enhances cell wall flexibility.	Promotes cell wall elasticity, allowing for better adaptation to changes in osmotic pressure during salt stress.	(Jensen et al., 2008; Mohnen, 2008)
Pectin Methylesterases	PME1, PME2	AT1G53840, AT1G53830	Demethylate pectin, modifying its properties and facilitating cross-linking.	Affect cell wall mechanics and influence cell adhesion, contributing to stress signaling and adaptation.	(Raiola et al., 2004)
Pectin Acetylesterases	AtPAE7	AT4G19410	Deacetylation of pectin, influencing cell wall properties.	Enhances cell wall stability and flexibility.	(Parniske et al., 1999)
	AtPAE5	AT3G09410	Modulates pectin structure for cell wall integrity	Improves osmotic adjustment and cell wall stability.	(Luhua et al., 2013)
	AtPAE3	AT4G19420	Participates in pectin modification during growth	Helps maintain structural integrity.	(Philippe et al., 2017)
Rhamnogalacturonan Synthesis	RGI1	AT3G24240	Synthesis of rhamnogalacturonan, a type of pectin that increases the complexity of the cell wall.	Helps retain water in the cell wall, preventing dehydration and maintaining turgor pressure.	(Jeon et al., 2023, 2024)

### 2.4 Secondary cell wall (lignin)

The secondary cell wall in plants mainly consists of cellulose, hemicellulose (primarily xylans and glucomannans), and lignin, along with various structural proteins and enzymes. Lignin is hydrophobic in nature, forms a substantial part of secondary cell wall biomass, and contributes to the rigidity of the plant (Scheller and Ulvskov, 2010). Both the quantity and composition of lignin varies across plant species. Lignin is structurally a complex polyphenolic polymer that integrates into cellulose and hemicellulose networks, providing mechanical strength, hydrophobicity, and rigidity to the secondary cell wall. It is primarily composed of three monolignols, i.e., coniferyl alcohol, ρ-coumaryl alcohol, and sinapyl alcohol, which are represented as guaiacyl (G), ρ-hydroxyphenyl (H), and syringyl (S), respectively, in the lignin polymer (Bonawitz and Chapple, 2010). The composition of these monolignols differs between species and cell types. The biosynthesis and acylation of lignin (monolignols) have been briefly described (Vanholme et al., 2019). Salt stress alters lignin concentration (**Table 5**). In response to salinity stress, maize cell walls decrease polysaccharides and arabinosyl feruloylation in arabinoxylan while increasing S-unit lignin polymers (Oliveira et al., 2020). Genotype-specific modifications in cell wall composition, including lignin and polysaccharide dynamics, play a critical role in determining plant tolerance to salt and drought stress by influencing wall plasticity and stress adaptability (Calderone et al., 2024). Salt stress increases lignin deposition in the root tracheary elements. A model was proposed in which increased lignin deposition limits ion uptake, enhances selective water transport through the symplastic pathway, reduces apoplast water flow, and restricts root growth (Sanchez-Aguayo et al., 2004). The change in lignin content and composition in response to abiotic and biotic stress has been briefly discussed by Moura et al. (2010), and recent studies suggest that selective downregulation of lignin can further improve salt stress tolerance by enhancing cell wall flexibility and facilitating better ion homeostasis in plants (Dokka et al., 2024).

**Table 5:** Genes involved in lignin biosynthesis and their role in salt stress response.

Gene Family	Gene Name/Distributed Gene Family	Function in Lignin Biosynthesis	Role under Salt Stress	Reference
Cinnamate-4-hydroxylase (C4H)	C4H	Catalyses the conversion of cinnamic acid to p-coumaric acid, a key step in the phenylpropanoid pathway leading to lignin biosynthesis.	Essential for lignin production, which reinforces the secondary cell wall, helping plants maintain structural integrity under salt-induced osmotic stress.	(Khatri et al., 2023)
Cinnamate-CoA Ligase	4CL	Catalyses the activation of p-coumaric acid to p-coumaroyl-CoA, a precursor in the lignin biosynthetic pathway.	Under salt stress, increased 4CL expression leads to enhanced lignin deposition, maintaining cell wall strength and preventing cell collapse or wilting due to osmotic imbalance.	(Teotia et al., 2019)
Laccase	LAC	Involved in oxidative polymerization of lignin precursors, forming lignin polymers in the secondary cell wall.	Laccase expression can increase under salt stress, leading to higher lignin deposition. This improves cell wall rigidity, offering protection against mechanical and osmotic stress.	(Wang et al., 2015; Bai et al., 2023; Zhu et al., 2023)
Peroxidase (PRX)	PRX	Catalyzes the polymerization of lignin monomers and participates in the oxidative stress response.	This strengthens cell walls, enhancing the plant’s ability to withstand osmotic and oxidative stress.	(Marjamaa et al., 2009; Gu et al., 2020; Peracchi et al., 2024)
Phenylalanine Ammonia-Lyase	PAL	Catalyzes the first step in the phenylpropanoid pathway, converting phenylalanine to cinnamic acid, which leads to lignin precursor synthesis.	Increased PAL expression under saline conditions enhances lignin production, which is critical for maintaining cell wall structure and stress tolerance.	(Wakabayashi et al., 2012; Amjad et al., 2024)
Cinnamoyl-CoA Reductase and Cinnamyl Alcohol Dehydrogenase	CCR, CAD	Reduces cinnamoyl-CoA esters to form cinnamaldehydes, which are critical intermediates for the production of lignin monomers.	Increased in response to salt stress. Higher levels of CCR and CAD help produce more lignin monomers, contributing to increased lignin content in cell walls.	(Giordano et al., 2014; Van Acker et al., 2017)
Caffeic Acid O-Methyltransferase	COMT	COMT methylates caffeic acid and 5-hydroxyferulic acid, contributing to the production of coniferyl and sinapyl alcohol.	Salt stress has been shown to increase the activity of these enzymes, particularly in the roots, where the protective role of lignin is most needed.	(Wagner et al., 2015; Eudes et al., 2017)
Ferulate 5-Hydroxylase	F5H	Hydroxylates ferulic acid to 5-hydroxyferulic acid, leading to the formation of sinapyl alcohol, one of the lignin monomers.		

Recent studies have highlighted the genetic and molecular regulation of lignin biosynthesis and its critical involvement in enhancing salt stress tolerance through diverse signaling and transcriptional networks. Mutation of histone acetyltransferase *GCN5* suppresses the expression of *chitinase like gene 1* (*CTL1*), which is essential for cellulose and lignin synthesis and salt tolerance in *Arabidopsis* (Zheng et al., 2019). The overexpression of *Superoxide Dismutase* (*SOD*), and *Ascorbate Peroxidase* (*APX*) in *Solanum tuberosum* L. induces cell wall lignification and promotes expression of different associated transcription factors during salt stress (Shafi et al., 2017). In apple, MYB46 enhances salt stress tolerance by increasing lignin accumulation and secondary cell wall synthesis and activates ABA-dependent and independent pathways that ultimately trigger various other defense responses (Chen et al., 2019). Overexpression of the sweet potato *SWPA4* gene in transgenic tobacco increases lignin deposition, hydrogen peroxide (H_2_O_2_) levels, and transcription of apoplast-related genes, which promote salt tolerance. Similarly, the two cultivars of soya bean with contrasting salt tolerance showed increased cell wall cellulose, pectin, and uronic acid under salt stress (An et al., 2014). Non-methylated uronic acid in the leaf cell wall contributes to salt resistance by reducing Na^+^ accumulation in plant tissues (Uddin et al., 2014). The dynamic regulation of lignin content and composition under salt stress reinforces cell wall integrity, modulates water and ion transport, and enhances stress resilience, highlighting its crucial role in plant adaptation to saline environments. These structural variations shape their remodeling responses, including differences in pectin de-esterification, lignification, and reactive oxygen species (ROS) signaling (Haas and Peaucelle, 2021).

### 2.5 Cell wall-associated proteins

The plant cell wall is not only vital for structural integrity but also for transducing environmental signals. Mechanical measurements using atomic force microscopy and Fourier-transform infrared analysis revealed that the cell wall structure is mechanically homogeneous; nevertheless, during the growth phase, stiffness increases on average as heterogeneity grows (Radotic et al., 2012). The assembly and regulation of cell wall proteins are essential for plant function, particularly in response to salt stress (**Table 6**). The heterotrimeric Gβ subunit AGB1 interacts with the receptor-like kinase FER and the peptide ligand RALF1 to modulate salt stress responses, highlighting a key signaling module in cell wall-associated salinity sensing (Yu and Assmann, 2018). LRXs further contribute to signaling pathways, interacting with RALF peptides, and coordinating growth regulation under stress conditions (Zhao et al., 2018; Wang and Gou, 2020). Structural proteins such as extensins play a vital role in maintaining cell wall architecture (Lamport et al., 2011). STELLO (STL) proteins in the Golgi facilitate CSC formation and may play a role in glycosylation, although their specific substrates are unknown (Zhang et al., 2016b). The CSC protein secreted from the endomembrane to the plasma membrane is altered depending on different aspects, such as the actin cytoskeleton via polymerization and depolymerization (Zhang et al., 2019), the pH of the endomembrane system (Luo et al., 2015), and interaction with proteins such as SHOU4 and TRANVIA (Polko et al., 2018; Vellosillo et al., 2021). Cortical microtubules coordinate the delivery of CSC to the plasma membrane (Gutierrez et al., 2009). Plants tolerate salt stress by altering their protein profile, i.e., salt-tolerant cultivars often express critical proteins more efficiently than salt-sensitive plants (Dissanayake et al., 2022), which shows that the quality of proteins is more important than quantity. Under salt stress, proteins including osmotin (*N. tabacum*) and gramin (*O. sativa*) play a vital role in maintaining cellular activities, while Na^+^ ion toxicity is alleviated by heat shock proteins (HSPs) and late embryonic abundant (LEA) proteins (Chourey et al., 2003; Derevyanchuk et al., 2016; Kumar et al., 2022). Despite the challenges in classifying cell wall proteins due to their diverse functions, recent classification has identified various categories, including those involved in metabolism, signaling, and structure (such as extensins, cellulose synthases, and pectin-modifying enzymes). Understanding the complex interplay of these proteins in stress perception and response is crucial for developing resilient crop varieties, as ongoing research seeks to explore the precise mechanisms by which the CWPs facilitate plant adaptation to environmental challenges.

**Table 6:** Roles of cell wall-associated proteins in salt stress response.

Gene Family	Gene Name/Distributed Gene Family	Gene ID	Function in Cell Wall Synthesis	Role under Salt Stress	Reference
Expansins (EXP)	EXP1, EXP7	AT1G69530, AT1G12560	Promote cell wall extension during growth and facilitates cell elongation.	Help maintain cell wall flexibility under salt stress, enabling cells to adjust to osmotic changes, which is critical for growth and adaptation to environmental stresses.	(Cosgrove, 2016; Ramakrishna et al., 2019)
Cellulases (CEL)	CEL1	AT1G70710	Degrade cellulose to enable cell wall turnover.	Modifies cellulose content under salt stress, to adjust cell wall structure and flexibility to cope with osmotic pressure during salt exposure.	(Shani et al., 2006)
Peroxidases (PRX)	PRX33	AT3G49110	Catalyze lignin polymerization and strengthen the secondary cell wall.	Peroxidase expression increases under salt stress, promoting lignin accumulation to strengthen the cell wall, improving resistance to osmotic stress, and preventing damage from oxidative stress.	(Daudi et al., 2012)
Glycosyltransferases (GTs)	CSLA9, IRX9	AT5G03760, AT2G37090	Involved in mannan and xylan biosynthesis, contributing to hemicellulose structure.	GTs are up-regulated under salt stress, enhancing the synthesis of polysaccharides and modifying cell wall composition, improving its mechanical properties to withstand osmotic stress.	(Davis et al., 2010; Meents et al., 2019; Barbut et al., 2024)
PME	PME1	AT4G12390	Modifies pectin properties by demethylating it, facilitating cell wall remodeling.	PMEs are crucial for adjusting cell wall composition under salt stress by maintaining elasticity and structural integrity, allowing cells to better tolerate osmotic imbalances.	(Gigli-Bisceglia et al., 2022; Sun et al., 2022)
Wall-Associated Kinases (WAKs)	WAK1, WAK2	AT1G21250, AT1G21270	Involved in signaling pathways related to cell wall integrity, linking external stimuli to cellular responses.	Mediate salt stress signaling, regulating cellular adaptation to osmotic imbalances by maintaining cell wall integrity and mediating stress-responsive gene expression.	(Decreux and Messiaen, 2005; Kohorn et al., 2009; Brutus et al., 2010)
Receptor-Like Protein Kinases (RLPKs)/RLKs	RLP23, BRI1	AT2G32680, AT4G39400	RLP23 participates in immunity, while BRI1 acts as a brassinosteroid receptor, modulating cell wall properties.	Regulate cell wall stiffness and adaptability, allowing for dynamic changes in cell structure to mitigate salt-induced damage.	(Bi et al., 2014; Albert et al., 2015; Kolomeichuk et al., 2023)
CrRLK1L (Catharanthus roseus RLK1-like)	FER, THE1, HERK1, ANJ	AT3G51550, AT5G54380, AT3G46290, AT5G59700	Regulate cell wall integrity and mechanosensing, influencing growth, ion transport, and ROS production.	FER senses cell wall changes during osmotic stress and activates stress response pathways, reinforcing cell wall structure. THE1 and HERK1 help adjust cell expansion, while ANJ participates in cell wall remodeling during salt stress.	(Lindner et al., 2012; Engelsdorf et al., 2019; Wang et al., 2021b; Gigli-Bisceglia et al., 2022; Jiang et al., 2024)
RALF (Rapid Alkalinization Factor)	–	–	RALF peptides interact with CrRLK1L receptors like FER to regulate cell wall-related processes and stress responses.	Modulates cell wall remodeling, pH balance in the apoplast, and the activation of stress-response genes, contributing to enhanced stress tolerance by stabilizing cell walls during salt stress.	(Zhang et al., 2023; Schoenaers et al., 2024)

## 3 Cell wall integrity is compromised during salt stress

The cell wall plays a crucial role in maintaining structural stability, regulating growth, and mediating stress responses. CWI can be compromised at various levels, resulting in altered physiological and biochemical responses that differ based on species, tissue type, and stress intensity. The CWI surveillance system monitors the condition of the cell wall, which is essential for stress perception and response (Vaahtera et al., 2019; Rui and Dinneny, 2020). Several cell wall sensors involved in CWI monitoring have been identified. Plants sense salinity at multiple cellular sites, primarily the plasma membrane, cell wall, and intracellular organelles (Zhu, 2016). Salinity stress response involves a large family of plasma membrane-localized and cell wall-associated Catharanthus roseus receptor-like-kinase-1-like (CrRLK1L) proteins, of which 17 members have been found in *Arabidopsis* (Lindner et al., 2012). These proteins interact with cell wall components, facilitating interactions between the cell wall and interior cellular processes (Feng et al., 2018). Other proteins, such as those from the wall-associated kinases (WAK) and leucine-rich repeat (LRR) receptor kinase families, are also thought to bind cell wall components and generate intracellular responses during cell wall changes (Herger et al., 2019; Lou et al., 2020). Perturbations in CWI can affect cellular turgor pressure, leading to shifts in membrane tension and cell wall stress. This, in turn, activates mechanosensitive channels in the plasma membrane, promoting ion accumulation and initiating downstream signaling processes (Hamann et al., 2004; Hamann, 2012; Bacete and Hamann, 2020). Consequently, the composition and integrity of the primary cell wall must be tightly monitored and regulated to support cell expansion and rapidly adapt to changing environmental conditions. In addition to the cell wall, cellular organelles such as chloroplasts and the ER are involved in stress sensing. Chloroplasts not only house essential photosynthetic proteins but also play a role in detecting salt stress, with mechanisms involving the regulation of ion transport and ROS production. The ER, which manages calcium homeostasis, also processes stress-related proteins, with specific proteins like ZmSep15-like-2 shown to mitigate salt-induced oxidative stress (Zhu et al., 2019).

Salt stress alters the lipid components of the plasma membrane, affecting tension and facilitating binding of Na^+^ by glycosyl inositol phosphorylceramide (GIPC) sphingolipids, crucial for salt-triggered Ca^2+^ influx (Jiang et al., 2019). In Arabidopsis, the salt stress pathway involves calcineurin B-like protein SOS3 (CBL4), CBL-interacting protein kinase SOS2 (CIPK24), and Na^+/^H^+^ antiporter SOS1, which work together to coordinate Na^+^ extrusion and mitigate toxicity (Yang and Guo, 2018; Song et al., 2024). During salt and osmotic stress, vacuoles play a crucial role in regulating Na+ levels through Na^+^/H^+^ exchange, driven by proton gradients formed by vacuolar H^+^-ATPases. These processes help maintain cellular ion balance, while stress-induced changes like turgor reduction and membrane tension alterations trigger complex biochemical signaling cascades (Nongpiur et al., 2020; Rui and Dinneny, 2020). Proteins such as QIAN SHOU KINASE (QSK1) rapidly localize to plasmodesmata in response to osmotic stress, though their specific role in these signaling pathways remains unclear (Grison et al., 2019). Membrane tension changes are also sensed by OSCA1, which may regulate cytosolic Ca^2+^ levels as part of the stress response (Yuan et al., 2014). Additionally, the RAF-SnRK2 pathway is activated, mediating key processes involved in ABA and osmotic stress responses, highlighting the intricate network of mechanisms plants use to adapt to saline environments (Boudsocq et al., 2004; Takahashi et al., 2020).

### 3.1 ROS and the cell wall in salt stress

Reactive oxygen species (ROS) are oxygen-derived molecules that function as both essential signaling mediators and potential inducers of cellular damage under stress conditions. The major forms of ROS include superoxide anion (·O_2_^-^), hydrogen peroxide (H_2_O_2_), hydroxyl radical (·OH), and singlet oxygen (^1^O_2_). Salinity triggers osmotic, ionic, and oxidative stress in plants (van Zelm et al., 2020). Initially, osmotic stress caused by decreasing water supply reduces cellular turgor, impacting cell expansion and tissue retraction. To retain cell integrity, plants actively adjust their cell walls to reinforce structure and restore osmotic potential (Zonia and Munnik, 2007). Mutants with impaired cell wall biosynthesis, such as *cesa6* and *mur4*, display pronounced growth inhibition under salt stress, underscoring the essential role of CWI in maintaining structural resilience and enabling adaptive responses to salinity (Zhang et al., 2016a; Zhao et al., 2020). Excessive Na^+^ accumulation causes ionic stress that disturbs enzymatic activity essential to metabolism and ion balance, which in turn affects the mechanical characteristics of cell walls, especially of pectin. Increased Na^+^ levels compete with Ca^2+^, disrupting pectin cross-linking, whereas salt stress activates pectin methyl esterases, affecting cell wall composition (Beauzamy et al., 2015; Gigli-Bisceglia et al., 2022). While ROS generated during salt stress can function as secondary messengers that allow peroxidases to remodel cell walls, excessive accumulation of ROS can cause oxidative damage and even plant death (Ma et al., 2012; Tenhaken, 2014). Thus, maintaining a balance of ROS levels is critical for plant survival in saline conditions. Mutants with deficient ROS regulation have revealed a complex relationship between plant ROS homeostasis, cell wall dynamics, and salt stress (Hong et al., 2020; Zhou et al., 2021). The synthesis of ROS during salt stress has both positive and negative impacts on plant growth. While high ROS can lead to oxidative damage and compromise cell integrity, low ROS can promote stress tolerance and cell wall remodeling (Liu et al., 2012). The balance between ROS generation and scavenging is crucial for maintaining CWI. Mutants with decreased ROS scavenging systems exhibit increased sensitivity to salinity (Hazman et al., 2015). Enzymes such as SOD and peroxidases help regulate oxidative stress. Furthermore, ROS-induced post-translational changes of cell wall components may influence the mechanical characteristics of the cell wall, influencing plant resilience under salt stress (Karkonen and Kuchitsu, 2015).

### 3.2 Microtubule dynamics under salt stress

Plant with decreased anisotropic growth under salt stress contain disrupted cellulose assembly and microtubule dynamics. Salt stress changes the dynamics of CSCs and microtubules in *Arabidopsis*, resulting in the removal of CesAs from the plasma membrane and microtubule depolymerization quickly after salt exposure (Komis et al., 2002; Wang et al., 2007; Gutierrez et al., 2009; Crowell et al., 2010). Moreover, the actin cytoskeleton significantly contributes to plant responses under salt stress, influencing cellulose synthase distribution and overall cell wall organization. However, after several hours of restoration of non-saline condition, microtubules reassemble and CesAs return to the membrane, indicating that plants possess adaptive mechanisms for cellulose synthesis during recovery from salt stress (Wang et al., 2007; Endler et al., 2015). The recovery of microtubule organization during salt stress depends on critical proteins like CC1 and CC2, which are essential components of CSCs. Mutants deprived of proper functioning of these proteins exhibit decreased growth, particularly in saline environments (Endler et al., 2015). Salt tolerance and CC1 function are impaired when microtubule-binding motifs are disrupted, as this prevents proper interaction between CC1 and microtubules, hindering cellular processes essential for stress adaptation (Kesten et al., 2022). While there is no direct animal tau protein homolog in plants, similarities in microtubule-binding properties suggest that plant proteins, such as CC1, may perform a similar role in regulating microtubule dynamics. Tau proteins in animals stabilize microtubules by binding to them, and it is hypothesized that salt stress might affect CC1, possibly through phosphorylation. This modification could alter the interaction between CC1 and microtubules, thereby impacting microtubule stability and cellular processes crucial for the plant’s response to salt stress (Mietelska-Porowska et al., 2014). Another key component of the CSC is KOR1, an endoglucanase found in the CSC that is internalized during salt stress and contribute to cellulose production (Nagashima et al., 2020). However, more research on KOR1 trafficking is needed, as pharmacological agents such as phenylarsine oxide and 1-butanol used to deduce its function also affect broader signaling pathways and may not specifically target KOR1 trafficking (McLoughlin et al., 2013; Gujas et al., 2017). Interestingly, phosphatidic acid (PA), associated with resistance to salt stress, functions in cellular signaling and may impact the localization of KOR1 (Zhang et al., 2012). Understanding this interaction is critical for determining plant responses to salt stress and controlling cellulose production.

Salt stress significantly disrupts cytoskeletal organization, which in turn affects CWI and plant tolerance mechanisms. Microtubule dynamics, in particular, are crucial in coordinating cellular responses to salinity. Chemical inhibitors like oryzalin (a microtubule depolymerizer) and taxol (a stabilizer) have been shown to markedly influence plant salt tolerance (Wang et al., 2007). Microtubule stability is maintained by proteins like MAP65-1, which enhances microtubule bundling, and its loss of function increases vulnerability to salt stress (Zhang et al., 2012). PA produced by phospholipase D, not only activates MAP65–1 but also interacts with MPK6, together contributing to microtubule organization and salt tolerance (Zhou et al., 2017). MPK6 further coordinates salt stress responses by linking cytoskeletal stability to broader signaling networks, including the SOS pathway (Yu et al., 2010). Additional microtubule-associated proteins, such as RIC1, facilitate microtubule reassembly after salt-induced disruption, while recently identified tropomyosin-like (TTL) proteins stabilize microtubules and interact with the CSC, directly linking cytoskeleton dynamics to cell wall biosynthesis and stress resilience (Kesten et al., 2022). Actin filaments are also critical, with salt stress-induced expression of actin depolymerizing factor 1 (ADF1) supporting filament remodeling, and loss of ADF1 leading to defective actin organization and reduced seedling survival (Wang et al., 2021a). These cytoskeletal adjustments are tightly integrated with CWI sensing, which relies on plasma membrane-localized receptors such as FER and other RLKs that detect wall perturbations and trigger Ca^2+^ influx during salt stress (Jose et al., 2020). The interaction of signaling modules like LRX3/4/5 with RALFs further highlights the complexity of CWI perception and its regulation under stress (Zhao et al., 2018). While these studies advance our understanding of the cytoskeleton–cell wall interface in salinity responses, the downstream intracellular signaling pathways, particularly those centered on MPK6, remain poorly understood. Elucidating these pathways will be key to developing targeted strategies for improving salt tolerance in crops.

The interplay between the cytoskeleton and CWI is crucial for plant adaptation to salt stress. Microtubule dynamics are linked to cellulose synthesis, while actin filaments also contribute to cell wall organization. Salt stress affects the organization and stability of both cytoskeletal components, leading to altered cell wall properties. For instance, ADF influences the distribution of CSC, and its expression is modulated by salt stress (Crowell et al., 2010; Wang et al., 2021a). The coordination between microtubules and actin is essential for maintaining cell shape and growth under saline conditions, highlighting the importance of cytoskeletal regulation in stress responses.

## 4 Cell wall remodeling under salt stress

### 4.1 Salt stress alters cell wall growth and extensibility

In plant cells, the cell wall and plasma membrane are kept intact through turgor pressure. The gap between them is filled with plasma membrane proteins such as Arabinogalactan proteins (AGPs), Wall-Associated Kinases (WAKs), Fasciclin-like arabinogalactan proteins (FLAs), and CesA complexes. These proteins maintain the delicate balance between cell wall extensibility and turgor pressure. This balance is critical for plant cell growth, development, and division, ensuring that plants can grow and adapt to their environments. Thus, the interaction of these components is vital to the life cycle of a plant, driving its growth and structural integrity (Wolf and Greiner, 2012; Watanabe et al., 2015).

**Figure 2:**
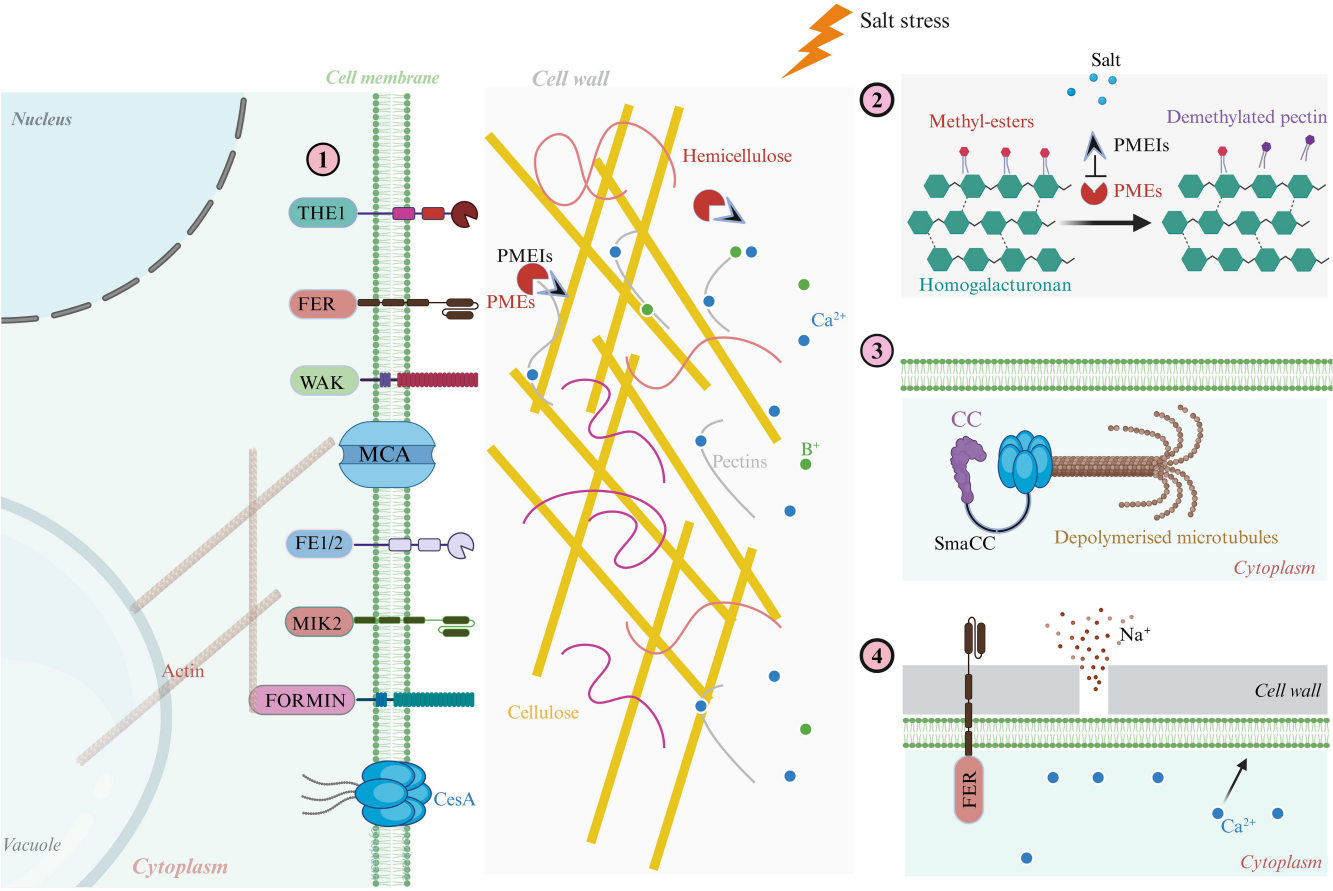
Cellular mechanisms of plant cell wall growth and extensibility under salt stress. This schematic illustrates how salt stress impairs cell wall extensibility by disrupting CWI, microtubule dynamics, ion balance, and calcium-pectin interactions, while also outlining mechanisms of recovery. (1) Under optimal conditions, key structural and signaling proteins such as AGPs, WAKs, FLAs protein, and CesAs, coordinate cell wall deposition and expansion, maintaining mechanical stability and enabling anisotropic growth. (2) PME modifies homogalacturonan domains, enhancing wall plasticity and promoting cell elongation. (3) Salt-induced ionic stress (notably Na^+^ influx) triggers cortical microtubule depolymerization, which impairs CesA trafficking and cellulose synthesis, compromising wall stiffness and directional expansion. (4) Upon stress alleviation, the receptor-like kinase FER modulates calcium–pectin cross-linking, restoring wall elasticity and supporting structural repair. Importantly, proteins such as FEI1/FEI2, MIK2, and FORMIN act as mechanosensors and regulators that bridge cytoskeletal organization with CWI maintenance, playing essential roles in signal transduction and cytoskeleton–cell wall coupling under salinity stress. These components collectively enable plants to perceive wall damage, transduce stress signals, and initiate adaptive remodeling responses crucial for survival in saline environments.

PME plays a significant role in cell wall extensibility and remodeling. It actively contributes to the loosening of the cell wall through the action of polygalacturonase, which degrades HG. This process is ultimately essential for cell adhesion and defense responses in plants. PME modifies the ionic content, cellular adhesion, and pH of plants under stress, influencing development through a mechanism called “acid growth” (Pelloux et al., 2007). Cell expansion and cell wall synthesis include four major processes, which are outlined in the review of Wolf and Greiner (2012). ROS are released when the cell wall senses changes. As a result of internal turgor pressure, the cell wall deforms, hydrates, and relaxes, and eventually, secretes new wall material. Cell wall extension in plants is primarily regulated by several key hormones. ROS are important players in cell wall development, as discussed by Karkonen and Kuchitsu (2015) (**Figure 2**). In the context of the cell wall, ROS act as agents that help with loosening and signaling various biological processes. They are produced by several enzymes (NADPH oxidases, quinone reductases, amine oxidases, lipoxygenases, oxalate oxidases, class III peroxidases), while various scavenger enzymes balance ROS levels, which is crucial for plant health. Auxin, a key plant hormone, promotes cell elongation by encouraging wall stretching and modification (Majda and Robert, 2018). However, when the cell wall stops expanding, it becomes rigid. This rigidity is due to the formation of strong connections known as di- and higher oligomer bridges, which are tightened further by ferulate coupling (Fry, 2004; Harris and Trethewey, 2009). H_2_O_2_ also contributes to this process, as it can inhibit cell wall growth through certain crosslinking reactions (Cona et al., 2006).

The cytoskeleton and associated proteins also play a critical role during plant cell division. Microtubule and actin filament structures are essential for this process, facilitating the synthesis of new cell walls through a specialized structure called the phragmoplast, which directs vesicles during cell division (Rasmussen et al., 2013). When plants face elevated salinity stress, they must adapt quickly to ensure proper cell expansion and resource utilization. Salt stress can lead to overaccumulation of Na^+^, causing K^+^ to efflux and increase ROS levels. This imbalance can lead to oxidative damage, disrupting turgor pressure and osmotic stability within the cells (Krasensky and Jonak, 2012; Zhang and Shi, 2013). One key player in this response is the CesA enzyme, which regulates cellulose synthesis to help manage the challenges posed by salt stress (Wang et al., 2016). Salt stress also destabilizes (depolymerizes) the microtubular structures of cells, causing CesA complexes in the membrane to become internalized (Paredez et al., 2006). A proposed model in which the CC protein interacts with microtubules and CesAs to maintain cellulose production and support plant growth during salt stress (Endler et al., 2015). BR, a class of plant hormones, also plays a vital role in this process. BR signaling may influence cell wall remodeling by regulating genes and enzymatic activities related to cell walls, particularly in grasses under stress (Rao and Dixon, 2017).

Additionally, proteins like RSA/MUR3/KAM1 help maintain the organization of actin microfilaments, enhancing salt tolerance by reducing ROS-induced damage (Li et al., 2013). During stress conditions, peroxidases utilize ROS in the apoplast to catalyze the cross-linking of phenolic compounds and glycoproteins in the cell wall, which helps to strengthen the wall and improve stress tolerance. This interaction reduces cell wall extensibility and limits cell expansion (Tenhaken, 2014). Interestingly, with prolonged salt exposure, cell wall extensibility can increase due to higher concentrations of ROS, specifically hydroxyl radicals. Enzymes such as xyloglucan-modifying enzymes and expansins in the apoplast can cleave glycosidic bonds in the cell wall, facilitating growth recovery (Tenhaken, 2014). Furthermore, the FER protein is essential for sensing salt stress and aiding cell recovery. In the absence of FER, cells may burst during recovery. It is believed that Na^+^ ions disrupt Ca^2+^ pectin cross-links in the cell wall, but FER helps balance Ca^2+^ levels by promoting cytosolic calcium, which is crucial for growth recovery (Feng et al., 2018). Understanding how these hormones and cellular mechanisms interact during salt stress can provide insights into enhancing plant resilience and improving agricultural practices (Mao et al., 2023).

### 4.2 Role of cutin and wax deposition in response to salt stress

The plant cuticle is primarily composed of cutin and waxes, forms a lipid-rich barrier over epidermal cell walls and plays a critical role in stress adaptation. Unlike the polysaccharide matrix of the inner wall, cuticular deposition is uniquely positioned to regulate non-stomatal water loss, ion exclusion, and mechanical protection traits vital under salt stress. Salt stress has been shown to induce transcriptional reprogramming of cuticle biosynthesis genes. For instance, studies in *Arabidopsis thaliana* and rice have demonstrated that salinity upregulates genes such as WIN1/SHN1, *CER1*, *LACS2*, and *WSD1*, which are involved in wax synthesis, transport, and polymerization (Liu et al., 2019; Tiika et al., 2025). Enhanced wax deposition under salinity improves leaf surface hydrophobicity and reduces passive ion influx, thereby contributing to ionic homeostasis.

Similarly, cutin biosynthesis is altered during salt exposure. The expression of genes such as *GPAT6*, *CYP86A2*, and *LCR* is responsive to salinity and modulates the ester-linked polyester matrix of the cuticle. Overexpression of *GPAT6* in *Solanum lycopersicum* was found to enhance both cutin monomer levels and salt stress tolerance by maintaining water potential and delaying ion toxicity (Petit et al., 2016). In addition to structural roles, cuticle-derived signals, such as fatty acid precursors and long-chain aldehydes, activate downstream ABA-dependent pathways or interact with ROS signaling, thereby integrating surface defense with internal stress responses (Lü et al., 2009). Collectively, these findings suggest that cuticle reinforcement through wax and cutin deposition is a critical but often underappreciated component of the plant’s salt stress response. By limiting water loss and serving as a biochemical shield, the epidermal barrier enhances plant survivability in saline environments. Incorporating cuticle modifications alongside cell wall remodeling provides a comprehensive understanding of plant surface adaptations to abiotic stress.

## 5 Anisotropic growth under salt stress

Anisotropic growth (the directional expansion of plant cells) is required for organ formation and overall plant architecture. A plant’s ability to grow plastically depends on the interaction of its cytoskeleton, plasma membrane, and cell walls. Although the relationship between the plasma membrane and the cytoskeleton has been extensively studied, the relationship between the plasma membrane and the cell wall itself is still unclear (Feraru et al., 2011; Liu et al., 2015). Cell wall orientation and mechanical properties are key determinants of anisotropic expansion. *In silico* modeling shows that pectin-cellulose interactions guide cell wall alignment and support elongation, particularly in structures like hypocotyls (Bou Daher et al., 2018). The orientation of cellulose fibers has been described as similar to a ‘hoop on a barrel’, highlighting its role in determining cell elongation. Cellulose fiber orientation varies significantly inside elongating cells. In epidermal cells, for example, cellulose fibers are more perpendicular on the inner faces, providing structural resistance to expansion, while on the outer faces, they exhibit a more random orientation to facilitate outward cell elongation (Chan et al., 2010, 2011; Crowell et al., 2011).

**Figure 3:**
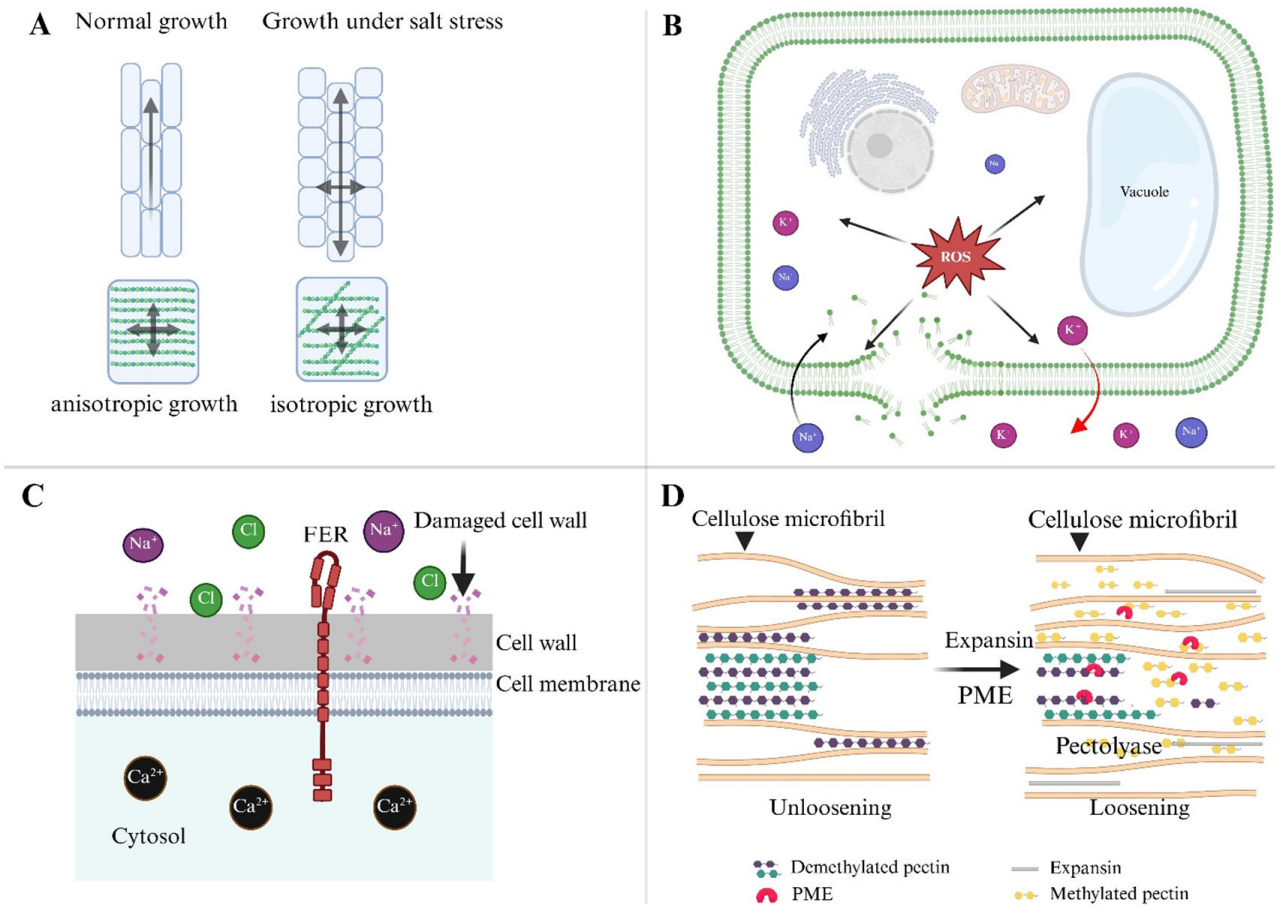
Mechanisms of anisotropic growth during salt stress in plants. **(A)** Under normal conditions, the cell wall cellulose and hemicellulose network support anisotropic growth, where cells elongate in a specific direction. In contrast, salt stress alters the orientation of cellulose fibers, resulting in isotropic growth, where cell expansion becomes less directional and more uniform across all axes. **(B)** Salt stress leads to an imbalance in ion homeostasis, with Na^+^ and K^+^ ions accumulating within the cell, disrupting the membrane’s integrity, and generating ROS. These ROS damage cell membranes and internal structures, inhibiting normal growth and causing cellular dysfunction. **(C)** The FER receptor plays a critical role in maintaining cell wall integrity during salt stress. FER interacts with extracellular signals and regulates ion homeostasis, helping to prevent excessive damage to the cell wall. By balancing Ca^2+^ and Cl^-^ levels, FER prevents premature cell bursting and supports anisotropic growth under stress. **(D)** Expansins are key enzymes in loosening the cell wall, enabling recovery after salt stress. They interact with pectin and cellulose components to relax the wall, facilitating cell expansion. The reorganization of microtubules and cellulose fibers, coupled with expansin activity, helps restore anisotropic growth patterns, allowing plants to adapt and recover from salt-induced growth inhibition.

The salt stress significantly disturbs the coordination mechanism, resulting in altered cell shape, disrupted tissue patterning, and reduced growth and yield (**Figure 3**). Excessive Na^+^ accumulation disrupts cellular homeostasis, disrupting the molecular and structural networks that control anisotropic growth. The cell wall, plasma membrane, and cytoskeleton form a dynamic interface that controls cell growth, but salt disturbs this interface on multiple levels. According to Baskin and Jensen (2013) individual cells have limited influence over their fate because they are integrated among tissues and organs. Under certain conditions, isotropic growth may result from stress-induced feedback processes. That challenges our understanding of cell wall dynamics. The cortical microtubules, which guide cellulose synthase movement, are highly sensitive to ionic imbalance (Wang et al., 2007). The dwarf mutant *any1* (anisotrophy1) exhibits reduced anisotropic development in roots, shoots, and trichomes, as well as decreased cell wall crystallinity and cellulose synthase complex velocity during expansion (Fujita et al., 2013). Most plants that are exposed to salt stress cease growing [216]. Anisotropic development under these conditions is significantly affected by modifications in cell wall architecture. As demonstrated by the function of SPIRAL1 (SPR1) in preventing anisotropic growth in *Arabidopsis thaliana*, the architecture of cortical microtubules is sensitive to ion homeostasis and can be harmed by salt stress (Nakajima et al., 2006). According to Buschmann and Borchers (2020), a study showing that cell expansion depends on signals from both apoplastic and cytoplasmic sources, salt stress can also alter intrinsic root development directions through microtubule depolymerization.

Furthermore, salinity alters pectin structure. Na^+^ binds to cell wall pectin, disrupting their charge balance and affecting wall porosity and elasticity, thus hindering expansion. The FER, a CWI sensor, helps maintain directional growth under stress. The *fer* mutants show abnormal root swelling and premature root initiation under salinity, indicating a failure in anisotropic expansion due to weakened wall integrity (Geng et al., 2013; Vaahtera et al., 2019). Similarly, *cesa* mutant, which impairs cellulose synthesis, reduces cell elongation even when microtubule orientation remains unaffected, highlighting the necessity of robust cell wall synthesis during salt stress (Fujita et al., 2013).

The actin cytoskeleton also plays a crucial role in anisotropic development under saline conditions. ARP2/3 complexes, which regulate actin meshwork formation, mediate vesicle trafficking of wall-modifying enzymes, while proteins like KINESIN-4A/FRA1 transport non-cellulosic materials along microtubules to support growth (Kong et al., 2015; Pratap Sahi et al., 2018). Disruption of these trafficking routes under salt stress further limits expansion.

On the other hand, FER seems to keep epidermal cells growing in an anisotropic form, preventing radial swelling and bursting. Importantly, certain molecular interventions can partially restore anisotropic growth under salt stress. Overexpression of *RhEXPA4*, a rose expansin gene, in *Arabidopsis* promotes root elongation and lateral root formation under saline conditions, emphasizing the role of wall-loosening proteins in maintaining directional growth (Lu et al., 2013). Nonetheless, the precise coordination of pectin de-methylesterification, xyloglucan remodeling, and PME activity during salt-induced stress remains underexplored. Finally, salt stress inhibits anisotropic growth via altering cell wall architecture, cytoskeletal dynamics, and CWI sensing. Understanding these systems is critical for developing salt-tolerant crops capable of maintaining directional growth and yield under challenging environments.

## 6 Conclusion and future perspectives

The plant cell wall serves as both a structural barrier and a dynamic sensor that orchestrates adaptive responses to environmental challenges such as salt stress. Salinity disrupts CWI, leading to impaired anisotropic growth by altering the deposition and organization of cellulose, hemicellulose, and pectin. These disruptions compromise wall extensibility, turgor balance, and cytoskeletal orientation, ultimately affecting plant development.

Despite advances in understanding cell wall remodeling under salt stress, the molecular mechanisms by which Na^+^ interferes with CWI perception and downstream signaling remain unclear. Receptor-like kinases such as FER and THE1 have emerged as key integrators of mechanical, hormonal, and immune pathways, mediating responses through ABA and JA signaling (Takahashi et al., 2020).

Future research should incorporate live-cell imaging, atomic force microscopy, and biomechanics to dissect how salt stress alters wall elasticity and polymer interactions at high resolution. Further exploration of calcium signaling, ROS dynamics, and their interactions with wall-associated kinases will deepen our mechanistic insights.

From a translational perspective, genome editing tools such as CRISPR/Cas9 and prime editing offer the potential to modify genes regulating wall biosynthesis, hormone crosstalk, and CWI sensing. Combining these with multi-omics approaches and systems biology will be critical to uncovering regulatory hubs that enable plants to maintain growth under salinity. A deeper mechanistic understanding of CWI under salt stress will ultimately facilitate the development of resilient, salt-tolerant crop varieties for sustainable agriculture.
